# Strategic rationing and freshness keeping of perishable products under transportation disruptions and demand learning

**DOI:** 10.1007/s40747-021-00492-w

**Published:** 2021-08-24

**Authors:** Shanshan Li, Yong He, Melissza Salling

**Affiliations:** 1 School of Finance, Nanjing Audit University, Nanjing, 211815 China; 2 School of Economics and Management, Southeast University, Nanjing, 210096 China

**Keywords:** Perishable products, Disruption management, Rationing, Freshness keeping, Demand learning

## Abstract

This paper considers a retailer who sells perishable fresh products directly to customers through an online channel and encounters a transportation disruption. Products shipped during the disruption period come with an uncontrollable delivery lead time, resulting in product quality degradation. To balance the compensation price provided to customers because of quality losses, the retailer might employ freshness-keeping efforts to reduce the quality loss during transportation. Therefore, it raises several fundamental questions for the retailer in mitigating the disruption. Is it always optimal to satisfy those customers who are willing to purchase during disruption? If it is profitable to fulfill orders along with an extra delivery lead time, and with a quality loss compensation, what is the optimal freshness-keeping effort? If it is preferable to deliberately create unsatisfied demand by announcing shortages (rationing) to customers, when is the optimal time to do so? To answer these questions, we first present the dynamics of post-disruption inventory and demand, taking into account the demand learning effect facilitated from negative word-of-mouth during disruption and the demand recovery after disruption ends. Afterward, we develop a model to achieve the optimal selling strategy for maximizing post-disruption profit, identifying the joint decision of the rationing period and freshness-keeping effort. Finally, by numerical analysis, three types of selling strategies are visually provided to hedge against disruptions of different lengths.

## Introduction

Unexpected events, such as natural disasters, are some of the major causes of distribution and production breakdowns in supply chains [1]. For example, during the COVID-19 pandemic, 94% of the Fortune 1000 companies have been undergone coronavirus-driven SC disruptions by March 2020 [2]. Fresh agricultural products, such as fruits, whose distribution systems mainly rely on just-in-time (JIT) manufacturing and delivery, are one of the most vulnerable and severely damaged industries. To control the spread of the COVID-19 virus, mandatory lockdowns are executed around half of the globe, causing shortages in the labour force and interruptions in logistics. As a direct consequence of such supply-side disruptions, JIT delivery cannot be fulfilled, and the delivery lead time becomes longer and uncontrollable, especially in the business model of online retailing.

Accordingly, an essential rationing question arises for the enterprise selling fresh products: whether to ship fresh products to the customers who purchase during the transportation disruption, or not?

On the one hand, due to the inherent perishability of fresh products, if the retailer decides to fulfill demand and arranges shipments during the transportation disruption, the quality might severely decay, when customers receive the fresh products after an uncontrollable delivery lead time. Nonetheless, the spoilage of fresh products can be prevented or alleviated throughout certain freshness-keeping efforts. For instance, considering that the quality deterioration rate mainly depends on the duration of logistics and the storage temperature, the retailer could employ refrigeration services during the process of transportation [3]. In addition, in Industry 4.0, with the support of technologies, such as RFID and time temperature indicator (TTI), and approaches of big data, the monitoring and controlling of product quality has been significantly enhanced [4]. Without freshness keeping, a penalty for quality loss is required from the retailer. The trade-off between the freshness-keeping price and the quality loss penalty leads to another critical question of what the optimal freshness-keeping effort is.

On the other hand, if the retailer deliberately chooses not to fulfill orders and announces stock-outs to the customers, i.e., to create rationing, both a short-term profit loss (i.e., lost sales) and a long-term market loss could be incurred. To be specific, by learning the negative word-of-mouth of the customers who have experienced stock-outs, the willingness of subsequent customers to place orders could be affected (hereinafter, “demand learning”) [5, 6]. In recent years, due to the emergence of e-commerce and social media platforms (such as Twitter, WeChat, etc.), the behavior of costumers can be disseminated and interacted with easier accesses in the digital era, thus facilitating the “demand learning” effect. For example, consumers can directly learn the previous behavior of others, through online product reviews [7]. Under the effect of “demand learning”, the future market potential could be reduced from a long-term aspect. Nonetheless, demand could be fully or partially recovered to the pre-disruption level, if some recovery policies, such as issuing discount coupons to the market, are implemented after the disruption ends.

In general, in view of the trade-offs between quality loss compensation, freshness-keeping price, lost-sale cost, market potential loss under demand learning, inventory cost, and market recovery cost, it is crucial for the enterprise to optimize the joint decision of freshness keeping and rationing, thus to maximize the post-disruption profit. This paper focuses on addressing the main questions mentioned previously.

In the existing literature, a lot of countermeasures have been proposed to cope with supply-side disruptions. For example, the pre-disruption tactics, focus on improving SC resilience, including inventory buffer, backup supply, transportation infrastructures, SC network design, etc. [8, 9]. From the perspective of post-disruption mitigation, the approaches such as coordination contracting, contingent sourcing, and service/production recovery, are established to alleviate the negative impact after the occurrence of supply disruptions [10]. However, the research is still in its infancy regarding the fresh produce industry, especially from the reactive aspects. In fact, the perfect proactive strategy is commonly impossible to be executed in practice or infeasible in economic terms [11]. Furthermore, in the context of fresh agriculture product retailing, the majority of enterprises are small medium (SMEs). For example, in Canada, small operations with less than 100 employees accounted for 94.1% of food and beverage processing establishments in 2016 [12]. The SMEs are often not well prepared to cope with disruptions, which emphasises the importance of designing effective strategies from post-disruption mitigation [13].

Capacity/inventory rationing is a well-utilized tactic for enterprises to control customers’ purchasing behaviour by adjusting the fulfill rate of demand and has been largely discussed in retailing [14]. Despite extensive attention on both rationing and disruption management, according to our best knowledge, there have been no studies found on designing reactive strategies in terms of freshness-keeping and rationing to mitigate transportation disruptions. Therefore, this paper attempts to fill this gap in the current literature. Noting that unlike in most papers on rationing [15], the purpose of creating unsatisfied demand in our paper, is not to facilitate panic buying among customers, but rather to balance the quality, quantity, time, and cost of delivering fresh products during the transportation disruption.

We contribute to transportation disruption management in the context of the fresh produce supply chain, as follows. A dynamic selling strategy is proposed to identify rationing time and freshness-keeping effort maximizing the retailer’s post-disruption profit, taking into account the following three disruption-related periods: disruption duration, post-disruption delivery lead time, and market recovery after the end of disruption. Customers’ interaction through information dissemination is also considered. By numerical analysis, we provide three types of strategies: “D&N”, “D”, and “N”, dependent on the lengths of rationing periods (i.e., the period during which the non-delivery strategy “N” is utilized to create unsatisfied demand). The corresponding freshness-keeping efforts during transportation are presented in closed form. Specific suggestions are provided for practitioners, guiding how to jointly adopt rationing periods and freshness-keeping efforts in accordance with various factors, including the selling and freshness-keeping prices of products, the cost and time of market recovery, the disruption length and delivery lead time, and the customers’ learning intensity towards the information of the previous period.

This paper is organized as follows: We briefly review the related literature in the next section. The following section describes and formulates the optimization problem. The optimal reactive strategy that incorporates rationing and freshness keeping maximizing the retailers’ post-disruption profit is presented in the next section. The following section gives a brief conclusion of the present paper.

## Literature review

The relevant literature mainly falls into two streams: supply-side disruption management of perishable product supply chain, and operational management regarding delivery lead time.

### Supply-side disruption management of perishable product supply chain

A fruitful of strategies have been proposed to hedge against supply-side disruptions, such as proactive strategies utilized before the occurrence of disruptions, reactive mitigation and recovery strategies to be implemented during and after disruption [16–19].

However, the extant research in the context of the perishable product supply chain is limited, and mainly focuses on the identification of risks and proactive tactics. For instance, as identified in [20], risks in the food supply chain could fall into multiple categories including product/service management risk, macro-level risk (e.g., natural disaster), demand management risk, supply management risk, and information management risk. Taking into consideration the interactions among diversified risks, [21] additionally presents a frame of evaluating major risks in food supply chains, through a blended grey-based Decision-Making Trial and Evaluation Laboratory model.

Together with an agri-food case study, [22] presents the designing of a robust multi-product supply chain network comprising several capacitated production facilities, distribution centres, and retailers, to hedge against disruptions generated from both demand-side and supply-side uncertainties. Based on a bi-objective mixed-integer programming model and a multiple sourcing policy, [23] also addresses the resilient design problem for a food supply chain to ensure business operations continuity in dealing with demand-side fluctuations and supply-side disruptions. A comprehensive framework that defines and considers resilience strategies in agri-food supply chains is provided in [24]. Recently, inspired by the COVID-19 pandemic, a few papers pay attention to pandemic-specific disruption management in food supply chains. Considering that the dearth of labour and truck drivers leads to food supply chain disruptions in affected regions, [25] develops a resilient and responsive public distribution system network through a simulated study. Through the method of fuzzy linguistic quantifier order weighted aggregation, [26] identifies and assesses the agriculture supply chain risks caused by disruptions. Various strategies, such as the adoption of industry 4.0 technologies, supply chain collaboration, and shared responsibility, are also correspondingly established for the practitioners to overcome the negative impact caused by risk events like the COVID-19 pandemic.

### Operational management regarding delivery lead time

Delivery lead time is mainly divided into two types: controllable and uncontrollable. The relevant literature regarding controllable delivery lead time mostly focuses on how to reduce the length or the uncertainty of delivery lead time from the operational direction, including production capacity [27], inventory policies, coordination among supply chain echelons [28], etc. For example, considering that customers are price and lead time sensitive, [29] investigates the optimal pricing, promised delivery lead time, supplier selection, and order allocation among the selected suppliers, to maximize the total profit of a multi-national pharmacological supply chain that comprises a retailer and multiple candidate capacity-constraint producers. With respect to uncontrollable delivery lead time, the majority of existing research focuses on examining how delivery lead time plays an important role in decisions such as inventory controlling, order placement, pricing, etc. [30–33].

Specific to the perishable product supply chain, quality decay is taken into consideration in the process of making decisions [34]. For instance, by formulating a mixed-integer linear programming model that positions stocks and allocates processes, [35] investigates the logistics network design problem under product quality decay and presents different network structures in accordance with the levels of decay.

The most relevant literature of our paper is the one incorporating disruption risks with delivery lead time. Considering that facilities serve customers based on delivery lead time and facilities’ capacity varies randomly due to disruptions, [36] presents a multi-period supply chain (SC) resilient network incorporating both mitigation and contingency strategies. In this paper, the mitigation strategy refers to fortifying warehouses against disruptions during the design phase of SC, and the contingency strategy stands for revising the decisions of assigning customers to SC facilities after disruptions occur. Taking a stochastic lead time and a penalty cost for delayed shipments in a single-vendor–single-buyer inventory model into account, [37] proposes an optimal vendor–buyer cooperative policy of reorder point, order quantity, and the number of shipments. Similarly, by extending the Newsvendor model with stock-out-based consumer switching behavior to include delivery lead time, [38] investigates the optimal decisions of retailer’s order quantity and manufacturer’s inventory level. Reference [39] extends the vendor–buyer problem by considering variable lead time.

Based on the above analysis, our paper differs from the existing literature in three dimensions. First, unlike most of the literature which focuses on proactively mitigating disruptions, we concentrate on reactively hedging against transportation disruptions after the occurrence of unexpected events, through rationing in conjunction with freshness-keeping effort. Freshness keeping is wildly recognized as an important measure for reducing fresh products’ quality decay during transportation [40]; however, little has been done to consider it as a contingency tool for alleviating unexpected transportation disruptions. Second, in addition to the disruption length which is commonly taken into account in disruption management, we also consider two types of time factors: the delivery lead time during transportation disruption and the post-disruption market recovery period. The recovery period is characterized based on two aspects: the recovery speed after disruption ends, and the lost demand that has incurred during disruption. Third, most literature assumes that customers are sensitive to quality, price, or time. In view of the negative word-of-mouth facilitated through customers’ interaction, we incorporate “demand learning” in the evaluation of post-disruption demand dynamics.

## Problem description and model formulation

Notations utilized in this paper are summarized in Table 1.

**Table 1:** Notations

Notations	Description
Decisions	
$t_{{\text{d}}}$	Entry and terminal time to create rationing under the strategies “D&N” and “N&D”
$u$	Freshness-keeping effort level
Parameters	
$c$	Unit production/growing cost
$c_{{\text{r}}}$	Unit recovery cost
$c_{{\text{f}}}$	Unit cost for each level of freshness-keeping effort per unit of time
$c_{{\text{h}}}$	Unit inventory holding cost per unit of time
$r$	Recovery speed of demand
$p$	Unit selling price of a fresh product
$d^{i} \left( {t, t_{{\text{d}}} } \right)$	Demand at time $t$ under policy $i$, when the delivery starts or ends at time $t_{d}$ during disruption
$I^{i} \left( {t, t_{{\text{d}}} } \right)$	Inventory level at time $t$ under policy $i$, when the delivery during disruption starts or ends at time $t_{{\text{d}}}$
$q\left( u \right)$	Quality of the fresh products, linked to freshness-keeping effort $u$
$T_{{\text{L}}}$	Delivery lead time during disruption
$T$	Length of the transportation disruption
$T_{\max }$	Time of the entire selling period
$k$	Quality decay rate of the fresh products
$d_{0}$	Demand rate before disruption occurs
$I_{0}$	Initial inventory at time 0
$\theta$	Learning intensity of customers
${\Pi }^{i}$	Retailer’s profit under the implementation of policy $i$

Following the basic setting of [6], we consider a retailer selling fresh products directly to customers via an online channel during a fixed selling period $\left[ {0, T_{\max } } \right]$. $d_{0}$ customers arrive at time $t$ and purchase online with price $p$, and will receive their products with a guaranteed quality “1”. Without loss of generality, the transit/delivery lead time is assumed to be zero in the absence of disruptions. To fulfill demands, the retailer stocks $I_{0}$ units of fresh products at the beginning of the selling cycle (i.e., at time ‘0’). We suppose that an unexpected event occurs at time ‘0’ and has a deterministic duration $T$. Affected by the event, the shipments realized at time $t \in \left[ {0, T} \right]$ cannot be delivered to customers instantly. That is, a transportation disruption with length $T$ happens.

If no countermeasures are adopted, the customers will receive fresh products with quality $q_{0}$ after a lead time $T_{{\text{L}}}$, as well as a quality-dependent compensation/refund (i.e., the penalty from the retailer’s perspective view). Following a widespread assumption in the existing literature [41], we consider an exponential decay rate in quality and formulate $q_{0}$ as

1 $$ q_{0} = {\text{e}}^{{ - kT_{{\text{L}}} }} , $$

where $0 \le q_{0} \le 1$. The parameter $k$ stands for the quality decay rate without freshness-keeping effort.

Facing the transportation interruption, the extended and uncontrollable delivery lead time, and the penalty for quality loss, the retailer considers two selling strategies as options to hedge against the disruption: “D&N” and “N&D”, based on different rationing periods. The dynamics of inventory and demand under these two strategies are discussed next.

### The dynamics of inventory and demand under “D&N”

Under the “D&N” strategy, the retailer decides to satisfy the first batch of customers placing orders during the transportation disruption. In other words, the products are delivered to the customers who arrive during the time interval $\left( {0, t_{{\text{d}}} } \right)$, under the effort $u$ for keeping fresh. Benefiting from the improved storage condition under freshness-keeping efforts, such as utilizing atmosphere storage rooms and refrigerated containers, customers will receive products with quality $q\left( u \right)$, as well as a refund (compensation) $\left( {1 - q\left( u \right)} \right)p$ because of partial quality loss. Noting that under the widely used technologies, like RFID and Time Temperature Indicators (TTIs), the product quality decay can be tracked in real time [35], thus making it convenient and widespread to implement the compensation service in accordance with quality:

2 $$ q\left( u \right) = {\text{e}}^{{ - k\left( {1 - u} \right)T_{{\text{L}}} }} , $$

where $0 \le u \le 1$. Particularly, when $u = 1$, it means that the retailer maintains the products’ quality level “1” during the entire transportation process by inputting the maximum freshness-keeping effort.

For the customers who arrive after time $t_{{\text{d}}}$, the retailer decides not to arrange deliveries and directly announces stock-outs. Therefore, at the termination of the period $\left( {t_{d} , t} \right)$, $\mathop \int \limits_{{t_{{\text{d}}} }}^{t} d_{1}^{D\& N} \left( {\tau ,t_{{\text{d}}} } \right){\text{d}}\tau$ customers have experienced stock-outs. Affected by the negative word-of-mouth facilitated from this portion of customers, the real-time demand rate at time $t$, i.e., $d_{1}^{{{\text{D}}\& {\text{N}}}} \left( {t,t_{{\text{d}}} } \right)$, drops over time thereafter [12], derived as

3 $$ d_{1}^{{{\text{D}}\& {\text{N}}}} \left( {t,t_{{\text{d}}} } \right) = d_{0} - \theta \mathop \int \limits_{{t_{{\text{d}}} }}^{t} d_{1}^{{{\text{D}}\& {\text{N}}}} \left( {\tau ,t_{{\text{d}}} } \right){\text{d}}\tau . $$

The parameter $\theta$ represents the learning intensity among customers, reflecting the tendency under which the customers’ consumption is affected by the previous period, $\theta \ge 0$. In particular, if $\theta = 0$, the negative word-of-mouth does not affect the subsequent customers’ purchasing behaviour. Solving Eq. (3), the demand dynamic can be further identified as

4 $$ d_{1}^{{{\text{D}}\& {\text{N}}}} \left( {t, t_{{\text{d}}} } \right) = d_{0} {\text{e}}^{{\theta \left( { t_{{\text{d}}} - t} \right)}} . $$

After the disruption ends at time $T$, the remaining demand can be satisfied immediately. The lost market potential (demand) $d_{0} - d_{1}^{{{\text{D}}\& {\text{N}}}} \left( {T,t_{{\text{d}}} } \right)$ could be gradually recovered with a certain price at a constant speed $r$ [11]. Accordingly, the demand in recovery can be derived as

5 $$ d_{2}^{{{\text{D}}\& {\text{N}}}} \left( {t, t_{{\text{d}}} } \right) = d_{1}^{{{\text{D}}\& {\text{N}}}} \left( {T, t_{{\text{d}}} } \right) + r\left( {t - T} \right). $$

The time when demand is fully recovered to the pre-disruption level can be described as follows:

6 $$ T_{r}^{{{\text{D}}\& {\text{N}}}} = T + \frac{{d_{0} - d_{1}^{{{\text{D}}\& {\text{N}}}} \left( {T, t_{{\text{d}}} } \right)}}{r} = T + \frac{{1 - {\text{e}}^{{\theta \left( { t_{{\text{d}}} - T} \right)}} }}{r}d_{0} . $$

Intuitively, if the reputation damage caused by stock-outs is devastating, or if it requires a long time to recover the lost demand per unit, the market potential might not be able to be fully recovered to the pre-disruption level at the end of the selling period. Thus, demand dynamics fall into two scenarios:

(i) Scenario 1 where $T_{r}^{{{\text{D}}\& {\text{N}}}} \le T_{\max }$: the market potential can be fully recovered, namely, “full recovery” in this study.
(ii) Scenario 2 where $T_{r}^{{{\text{D}}\& {\text{N}}}} > T_{\max }$: the market potential cannot be fully recovered, namely, “partial recovery”.

**Fig. 1:**
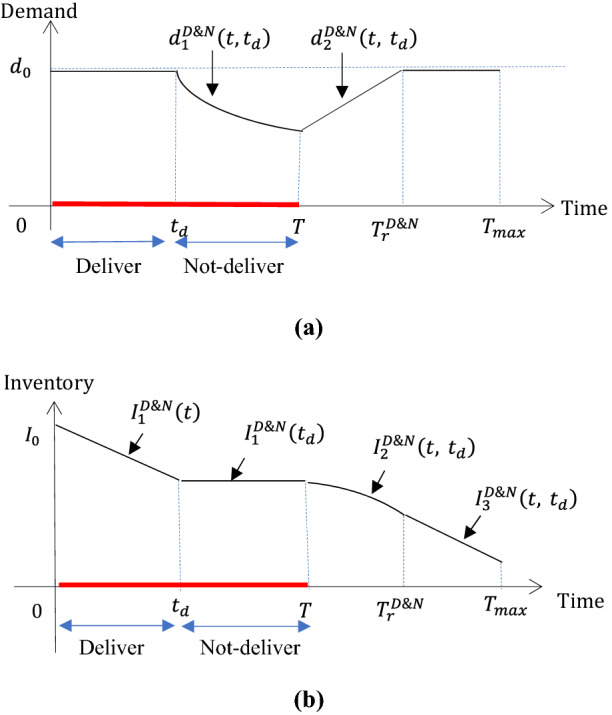
The dynamics of demand (**a**) and inventory (**b**) under the “D&N” strategy, in Scenario 1 where $T_{r}^{{{\text{D}}\& {\text{N}}}} \le T_{{{\text{max}}}}$

Figure 1a depicts the demand dynamics for Scenario 1. Based on Fig. 1a, we next discuss how the post-disruption inventory changes over time in Scenario 1. As shown in Fig. 1b, inventory is consumed at the speed $d_{0}$ (demand rate) during the first phase $\left( {0,t_{{\text{d}}} } \right)$. During the second phase $\left( {t_{{\text{d}}} , T} \right)$, the retailer chooses to announce a stock-out other than to arrange deliveries, thus, inventory maintains at a constant level. Then, inventory drops at the speeds of $d_{2}^{{{\text{D}}\& {\text{N}}}} \left( {t, t_{{\text{d}}} } \right)$ and $d_{0}$ during the time intervals $\left( {T, T_{r}^{{{\text{D}}\& {\text{N}}}} } \right) \cup \left( {T_{r}^{{{\text{D}}\& {\text{N}}}} , T_{{{\text{max}}}} } \right)$. To sum up, the inventory and demand dynamics under the strategy “D&N” in Scenario 1 can be specified in Table 2.

**Table 2:** The functions of demand and inventory under the selling strategies “D&N” and “N&D”

Cases			Demand and inventory functions in each case				
1–1	Under D&N	$T_{r}^{{{\text{D}}\& {\text{N}}}} \le T_{\max }$	Time interval	$\left[ {0,t_{{\text{d}}} } \right]$	$\left[ {t_{{\text{d}}} ,T} \right]$	$\left[ {T,T_{r}^{{{\text{D}}\& {\text{N}}}} } \right]$	$[T_{r}^{{{\text{D}}\& {\text{N}}}} ,T_{\max }$]
			$d^{{{\text{D}}\& {\text{N}}}}$	$d_{0}$	$d_{1}^{{{\text{D}}\& {\text{N}}}} \left( {t,t_{{\text{d}}} } \right)$	$d_{2}^{{{\text{D}}\& {\text{N}}}} \left( {t, t_{{\text{d}}} } \right)$	$d_{0}$
			$I^{{{\text{D}}\& {\text{N}}}}$	$I_{1}^{{{\text{D}}\& {\text{N}}}} \left( t \right)$	$I_{1}^{{{\text{D}}\& {\text{N}}}} \left( {t_{{\text{d}}} } \right)$	$I_{2}^{{{\text{D}}\& {\text{N}}}} \left( {t, t_{{\text{d}}} } \right)$	$I_{3}^{{{\text{D}}\& {\text{N}}}} \left( {t, t_{{\text{d}}} } \right)$
1–2		$T_{r}^{{{\text{D}}\& {\text{N}}}} > T_{\max }$	Time interval	$\left[ {0,t_{{\text{d}}} } \right]$	$\left[ {t_{{\text{d}}} ,T} \right]$	$\left[ {T,T_{\max } } \right]$	
			$d^{{{\text{D}}\& {\text{N}}}}$	$d_{0}$	$d_{1}^{{{\text{D}}\& {\text{N}}}} \left( {t,t_{{\text{d}}} } \right)$	$d_{2}^{{{\text{D}}\& {\text{N}}}} \left( {t, t_{{\text{d}}} } \right)$	
			$I^{{{\text{D}}\& {\text{N}}}}$	$I_{1}^{{{\text{D}}\& {\text{N}}}} \left( t \right)$	$I_{1}^{{{\text{D}}\& {\text{N}}}} \left( {t_{{\text{d}}} } \right)$	$I_{2}^{{{\text{D}}\& {\text{N}}}} \left( {t, t_{{\text{d}}} } \right)$	
2–1	Under N&D	$T_{r}^{{{\text{N}}\& {\text{D}}}} \le T_{\max }$	Time interval	$\left[ {0,t_{d} } \right]$	$\left[ {t_{d} ,T} \right]$	$\left[ {T,T_{r}^{{{\text{N}}\& {\text{D}}}} } \right]$	$[T_{r}^{{{\text{N}}\& {\text{D}}}} ,T_{\max }$]
			$d^{{{\text{N}}\& {\text{D}}}}$	$d_{1}^{{{\text{N}}\& {\text{D}}}} \left( t \right)$	$d_{1}^{{{\text{N}}\& {\text{D}}}} \left( {t_{{\text{d}}} } \right)$	$d_{2}^{{{\text{N}}\& {\text{D}}}} \left( {t, t_{{\text{d}}} } \right)$	$d_{0}$
			$I^{{{\text{N}}\& {\text{D}}}}$	$I_{0}$	$I_{1}^{{{\text{N}}\& {\text{D}}}} \left( {t, t_{{\text{d}}} } \right)$	$I_{2}^{{{\text{N}}\& {\text{D}}}} \left( {t, t_{{\text{d}}} } \right)$	$I_{3}^{{{\text{N}}\& {\text{D}}}} \left( {t, t_{{\text{d}}} } \right)$
2–2		$T_{r}^{{{\text{N}}\& {\text{D}}}} > T_{\max }$	Time interval	$\left[ {0,t_{{\text{d}}} } \right]$	$\left[ {t_{{\text{d}}} ,T} \right]$	$\left[ {T,T_{\max } } \right]$	
			$d^{{{\text{N}}\& {\text{D}}}}$	$d_{1}^{{{\text{N}}\& {\text{D}}}} \left( t \right)$	$d_{1}^{{{\text{N}}\& {\text{D}}}} \left( {t_{{\text{d}}} } \right)$	$d_{2}^{{{\text{N}}\& {\text{D}}}} \left( {t, t_{{\text{d}}} } \right)$	
			$I^{{{\text{N}}\& {\text{D}}}}$	$I_{0}$	$I_{1}^{{{\text{N}}\& {\text{D}}}} \left( {t, t_{{\text{d}}} } \right)$	$I_{2}^{{{\text{N}}\& {\text{D}}}} \left( {t, t_{{\text{d}}} } \right)$	

For notational simplicity, we omit the time indices $t$ and $t_{{\text{d}}}$ of $I^{{{\text{N}}\& {\text{D}}}} \left( {t, t_{{\text{d}}} } \right)$ $d^{{{\text{N}}\& {\text{D}}}} \left( {t, t_{{\text{d}}} } \right)$, $I^{{{\text{D}}\& {\text{N}}}} \left( {t, t_{{\text{d}}} } \right)$, and $d^{{{\text{D}}\& {\text{N}}}} \left( {t, t_{{\text{d}}} } \right)$ in Table 2.

Following the above discussion for Scenario 1, the inventory and demand dynamics of Scenario 2 can be similarly achieved in Table 2.

### The dynamics of inventory and demand under “N&D”

Opposite to “The dynamics of inventory and demand under “D&N””, under the “N&D” strategy, the retailer chooses to announce stock-outs to the customers placing orders at the first phase $\left( {0, t_{{\text{d}}} } \right)$, and satisfies the second batch of customers arriving during $\left( {t_{{\text{d}}} , T} \right)$. Thus, similar to (3), the real-time demand during the first phase drops and can be derived as

7 $$ d_{1}^{{{\text{N}}\& {\text{D}}}} \left( t \right) = d_{0} - \theta \mathop \int \limits_{0}^{t} d_{1}^{{{\text{N}}\& {\text{D}}}} \left( \tau \right){\text{d}}\tau , $$

where $\mathop \int \nolimits_{0}^{t} d_{1}^{{{\text{N}}\& {\text{D}}}} \left( \tau \right){\text{d}}\tau$ calculates the number of customers who have experienced stock-outs during the time interval $\left( {0, t} \right)$. Solving (7), $d_{1}^{{{\text{N}}\& {\text{D}}}} \left( t \right)$ is specified as

8 $$ d_{1}^{{{\text{N}}\& {\text{D}}}} \left( t \right) = d_{0} {\text{e}}^{ - \theta t} . $$

In the second phase $\left( {t_{{\text{d}}} , T} \right)$, the retailer terminates rationing and ships products to the remaining demand. No customer undergoes stock-out thereafter. Thus, demand maintains at the constant rate $d_{1}^{{{\text{N}}\& {\text{D}}}} \left( {t_{{\text{d}}} } \right)$ during this sub-phase and will be recovered to $d_{0}$ at time $T_{r}^{{{\text{N}}\& {\text{D}}}}$. Similar to (5)–(6), the real-time demand rate in recovery and the critical time when the demand recovery is completed can be described as

9 $$ d_{2}^{{{\text{N}}\& {\text{D}}}} \left( {t, t_{{\text{d}}} } \right) = d_{1}^{{{\text{N}}\& {\text{D}}}} \left( {t_{{\text{d}}} } \right) + r\left( {t - T} \right), $$

10 $$ T_{r}^{{{\text{N}}\& {\text{D}}}} = T + \frac{{d_{0} - d_{1}^{{{\text{N}}\& {\text{D}}}} \left( {t_{{\text{d}}} } \right)}}{r} = T + \frac{{d_{0} - d_{0} {\text{e}}^{{ - \theta t_{{\text{d}}} }} }}{r}. $$

**Fig. 2:**
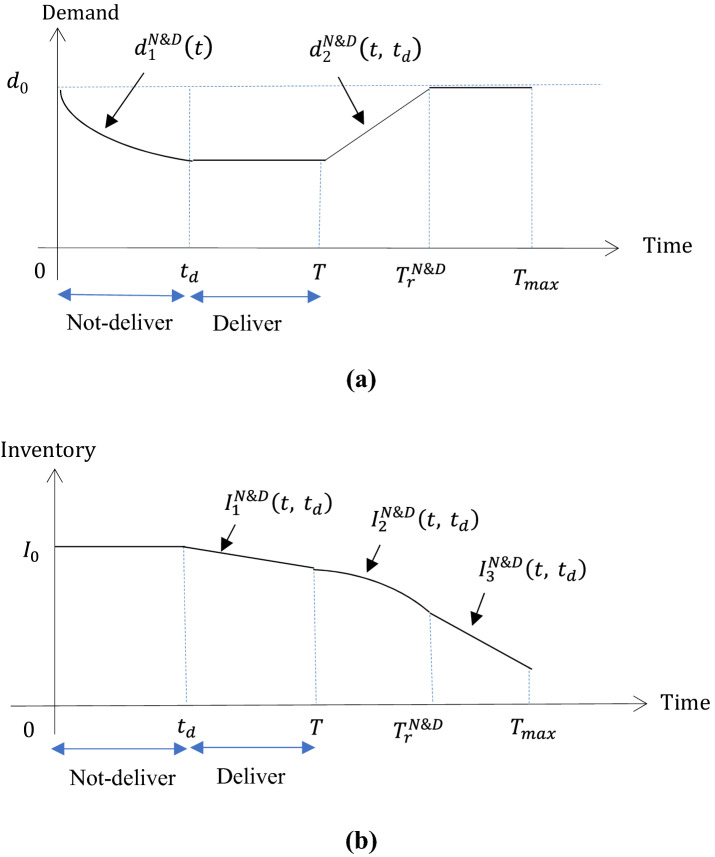
The dynamics of demand (**a**) and inventory (**b**) under the “N&D” strategy, in Scenario 1 where $T_{r}^{{{\text{N}}\& {\text{D}}}} \le T_{\max }$

Like Figs. 1, 2 depicts the dynamics of inventory and demand for Scenario 1 under the implementation of the “N&D” strategy.

Repeating the discussion in “The dynamics of inventory and demand under “D&N””, we can similarly identify the post-disruption demand and inventory dynamics in Scenario 2 (i.e., partial recovery), under “N&D”. The expressions are summarized in Table 2.

Where $d_{1}^{{{\text{D}}\& {\text{N}}}} \left( {t,t_{{\text{d}}} } \right)$, $d_{2}^{{{\text{D}}\& {\text{N}}}} \left( {t, t_{{\text{d}}} } \right)$ and $T_{r}^{{{\text{D}}\& {\text{N}}}}$ are given in (4)–(6). $d_{1}^{{{\text{N}}\& {\text{D}}}} \left( t \right)$, $d_{2}^{{{\text{N}}\& {\text{D}}}} \left( {t, t_{{\text{d}}} } \right)$ and $T_{r}^{{{\text{N}}\& {\text{D}}}}$ are given in (8)–(10).

11 $$ \begin{gathered} I_{1}^{{{\text{D}}\& {\text{N}}}} \left( t \right) = I_{0} - d_{0} t,\;I_{2}^{{{\text{D}}\& {\text{N}}}} \left( {t, t_{{\text{d}}} } \right) = I_{1}^{{{\text{D}}\& {\text{N}}}} \left( {t_{{\text{d}}} } \right) - \mathop \int \limits_{T}^{t} d_{2}^{{{\text{D}}\& {\text{N}}}} \left( {\tau , t_{{\text{d}}} } \right){\text{d}}\tau , \hfill \\ I_{3}^{{{\text{D}}\& {\text{N}}}} \left( {t, t_{{\text{d}}} } \right) = I_{2}^{{{\text{D}}\& {\text{N}}}} \left( {T_{r}^{{{\text{D}}\& {\text{N}}}} , t_{{\text{d}}} } \right) - \mathop \int \limits_{{T_{r}^{{{\text{D}}\& {\text{N}}}} }}^{t} d_{0} {\text{d}}\tau . \hfill \\ \end{gathered} $$

12 $$ \begin{gathered} I_{1}^{{{\text{N}}\& {\text{D}}}} \left( {t, t_{{\text{d}}} } \right) = I_{0} - \mathop \int \limits_{{t_{{\text{d}}} }}^{t} d_{1}^{{{\text{N}}\& {\text{D}}}} \left( {t_{{\text{d}}} } \right){\text{d}}\tau ,\;I_{2}^{{{\text{N}}\& {\text{D}}}} \left( {t, t_{{\text{d}}} } \right) = I_{1}^{{{\text{N}}\& {\text{D}}}} \left( {T, t_{{\text{d}}} } \right) - \mathop \int \limits_{T}^{t} d_{2}^{{{\text{N}}\& {\text{D}}}} \left( {\tau , t_{d} } \right){\text{d}}\tau , \hfill \\ I_{3}^{{{\text{N}}\& {\text{D}}}} \left( {t, t_{{\text{d}}} } \right) = I_{2}^{{{\text{N}}\& {\text{D}}}} \left( {T_{r}^{{{\text{N}}\& {\text{D}}}} , t_{{\text{d}}} } \right) - \mathop \int \limits_{{T_{r}^{{{\text{N}}\& {\text{D}}}} }}^{t} d_{0} {\text{d}}\tau . \hfill \\ \end{gathered} $$

### Mathematical representation of optimal mitigation strategies

By implementing the “N&D” and “D&N” strategies, the retailer achieves profits $\Pi^{{{\text{N}}\& {\text{D}}}}$ and $\Pi^{{{\text{D}}\& {\text{N}}}}$ during the entire selling period $\left( {0, T_{\max } } \right)$.

13 $$ \begin{aligned} \Pi^{{{\text{D}}\& {\text{N}}}} & = p\mathop \int \limits_{0}^{{T_{\max } }} d^{{{\text{D}}\& {\text{N}}}} \left( {t, t_{{\text{d}}} } \right){\text{d}}t - c_{{\text{f}}} uT_{{\text{L}}} \mathop \int \limits_{0}^{{t_{{\text{d}}} }} d_{0} {\text{d}}t - \left( {1 - q\left( u \right)} \right)p\mathop \int \limits_{0}^{{t_{{\text{d}}} }} d_{0} {\text{d}}t \\ & \quad - c_{{\text{h}}} \mathop \int \limits_{0}^{{T_{\max } }} I^{{{\text{D}}\& {\text{N}}}} \left( {t, t_{{\text{d}}} } \right){\text{d}}t - c_{{\text{r}}} \left[ {d_{0} - d_{1}^{{{\text{D}}\& {\text{N}}}} \left( {T, t_{{\text{d}}} } \right)} \right], \\ \end{aligned} $$

14 $$ \begin{aligned} \Pi^{{{\text{N}}\& {\text{D}}}} & = p\mathop \int \limits_{0}^{{T_{\max } }} d^{{{\text{N}}\& {\text{D}}}} \left( {t, t_{{\text{d}}} } \right){\text{d}}t - c_{{\text{f}}} uT_{{\text{L}}} \mathop \int \limits_{{t_{{\text{d}}} }}^{T} d_{1}^{{{\text{N}}\& {\text{D}}}} \left( {t_{{\text{d}}} } \right){\text{d}}t \\ & \quad - \left( {1 - q\left( u \right)} \right)p\mathop \int \limits_{{t_{{\text{d}}} }}^{T} d_{1}^{{{\text{N}}\& {\text{D}}}} \left( {t_{{\text{d}}} } \right){\text{d}}t - c_{{\text{h}}} \mathop \int \limits_{0}^{{T_{\max } }} I^{{{\text{N}}\& {\text{D}}}} \left( {t, t_{{\text{d}}} } \right){\text{d}}t - c_{{\text{r}}} \left[ {d_{0} - d_{1}^{{{\text{N}}\& {\text{D}}}} \left( {t_{{\text{d}}} } \right)} \right], \\ \end{aligned} $$

where $\Pi^{{{\text{N}}\& {\text{D}}}} , \Pi^{{{\text{D}}\& {\text{N}}}} \ge 0$. In (13), the second term presents the freshness-keeping cost incurred during the process of delivering the products to the customers in the time interval $\left( {0, t_{{\text{d}}} } \right)$. The third represents the refund that the retailer has to pay the customers for the product quality loss. The fourth term describes the inventory holding cost. The last stands for the market recovery cost. The interpretation of (14) is similar to (13). The demand and inventory functions are given in Table 2.

Based on the above evaluation of the retailer’s profit, the optimal reactive selling strategy to hedge against the transportation interruption can be formulated as follows:

15 $$ \begin{gathered} \{ u^{*} , t_{{\text{d}}}^{*} \} \in \arg \mathop {\max }\limits_{{\left\{ {u, t_{{\text{d}}} } \right\} }} \left( {\Pi^{{{\text{D}}\& {\text{N}}}} , \Pi^{{{\text{N}}\& {\text{D}}}} } \right), \hfill \\ {\text{where}}\;0 < t_{{\text{d}}} \le T\;{\text{and}}\;0 \le u \le 1. \hfill \\ \end{gathered} $$

Model (15) can be decomposed into two sub-questions:

Q1: $\{ u^{*} , t_{{\text{d}}}^{*} \} \in \arg \mathop {\max }\limits_{{\left\{ {u, t_{{\text{d}}} } \right\} }} \Pi^{{{\text{D}}\& {\text{N}}}}$ and $\{ u^{*} , t_{{\text{d}}}^{*} \} \in \arg \mathop {\max }\limits_{{\left\{ {u, t_{{\text{d}}} } \right\} }} \Pi^{{{\text{N}}\& {\text{D}}}}$. Firstly, we investigate the optimal point in time when to terminate and start deliveries (i.e., the entry and terminal time of rationing) and the optimal freshness-keeping effort in the process of transportation, for the strategies “D&N” and “N&D”, respectively.

Q2: Comparing $\Pi^{{{\text{D}}\& {\text{N}}}} \{ u^{*} , t_{{\text{d}}}^{*} \}$ with $\Pi^{{{\text{N}}\& {\text{D}}}} \{ u^{*} ,{ }t_{{\text{d}}}^{*} \}$, the optimal strategy type (“N&D” or “D&N”) is determined.

By addressing Q1 and Q2, the optimal selling strategy is determined from Model (15), identifying the optimal strategy type and the corresponding optimal joint decision of rationing time and freshness-keeping effort. In particular, if $t_{{\text{d}}}^{*} = T$ under the “D&N” strategy, it means that it is superior for the retailer to ship the products to all customers during the disruption, even though the deliveries might come with extra freshness-keeping efforts and penalties for quality deterioration. In this study, we refer to the “D&N” strategy with $t_{{\text{d}}}^{*} = T$ as the pure full-delivery strategy “D”. On the contrary, if $t_{{\text{d}}}^{*} = T$ under the “N&D” strategy, no delivery is arranged to any customers during disruption. In other words, the retailer chooses to announce stock-outs to all customers. Thus, the strategy “N&D” with $t_{{\text{d}}}^{*} = T$ is named as the pure non-delivery strategy “N” in the following. Table 3 summarizes the strategies derived in this paper.

**Table 3:** Strategies overview

Strategies	Decisions		Specification
	$\left( {0, t_{{\text{d}}}^{*} } \right)$	$\left( {t_{{\text{d}}}^{*} , T} \right)$	
“D&N”	Deliver	Not-deliver	$0 < t_{{\text{d}}}^{*} < T$
“N&D”	Not-deliver	Deliver	$0 < t_{{\text{d}}}^{*} < T$
“D”	Deliver		$t_{{\text{d}}}^{*} = T$ under “D&N”, or $t_{{\text{d}}}^{*} \approx 0$ under “N&D”
“N”	Not-deliver		$t_{{\text{d}}}^{*} \approx 0$ under “D&N”, or $t_{{\text{d}}}^{*} = T$ under “N&D”

With the optimal joint decision $\{ u^{*} , t_{{\text{d}}}^{*} \}$ from Model (15), we answer the research questions raised earlier: whether to create unsatisfied demand on purpose or not? If yes, when is the optimal time to start or end the announcement of stock-outs to customers? If not, what the optimal freshness-keeping effort is, to reduce fresh products’ quality loss.

## The optimal selling strategy

In this section, we explore $\{ u^{*} ,{ }t_{{\text{d}}}^{*} \}$ for the optimization problem (15). According to the profit functions (13)–(14), we find that the $u^{*}$ maximizing the profits $\Pi^{{{\text{D}}\& {\text{N}}}}$ and $\Pi^{{{\text{N}}\& {\text{D}}}}$ are independent with $t_{{\text{d}}}^{*}$. Thus, we next determine the optimal joint decision in two steps. Step1: We calculate $u^{*}$ for Q1 in “The optimal $u^{*}$”. Step 2: Given $u^{*}$, we explore $t_{{\text{d}}}^{*}$ for Q1 and Q2, in “The optimal $t_{{\text{d}}}^{*}$”.

### The optimal $u^{*}$

By solving (15), $u^{*}$ is achieved in Proposition 1.

#### Proposition 1

*The retailer’s optimal freshness-keeping effort is determined as* $u^{*} = 1$ *if*

16 $$ \left( {1 - {\text{e}}^{{ - kT_{{\text{L}}} }} } \right)p - c_{{\text{f}}} T_{{\text{L}}} > 0. $$

*Otherwise,* $$u^{*} = 0$$*.*

#### Proof

See the Appendix.

As indicated in Proposition 1, the optimal freshness-keeping effort only occurs at the boundaries. The economic interpretation for the condition under which $u^{*} = 1$ should be implemented (i.e., (16)) is as follows. It is preferable for the retailer to maintain all the products at the quality level “1” by inputting the maximum freshness-keeping effort during the entire transportation process, if the selling price of the fresh products is high, to be specific, exceeding a threshold $\frac{{c_{{\text{f}}} T_{{\text{L}}} }}{{\left( {1 - {\text{e}}^{{ - kT_{L} }} } \right)}}$.

Furthermore, by discussing condition (16), we shed further light on how to change the freshness-keeping effort in accordance with the delivery lead time and the freshness-keeping price.

#### Corollary 1

(i) *If* $$kp - c_{{\text{f}}} > 0$$*:* $$u^{*} = 1 $$ *when* $$T_{{\text{L}}} < A$$*, and* $$u^{*} = 0$$ *when* $$T_{{\text{L}}} \ge A$$*.*
(ii) *If* $$kp - c_{{\text{f}}} < 0$$*:* $$u^{*} = 0$$*.*

*Where* $A$ *is defined as the positive solution of equation* $\left( {1 - {\text{e}}^{{ - kT_{{\text{L}}} }} } \right)p - c_{{\text{f}}} T_{{\text{L}}} = 0$ *with respect to variable* $T_{{\text{L}}}$*.*

#### Proof

See the Appendix.

Corollary 1 suggests that the retailer makes the maximum freshness-keeping effort during transportation if neither the freshness-keeping price nor the delivery lead time will be large, i.e., $c_{{\text{f}}} < kp$ and $T_{{\text{L}}} < A$. It is also worth noting that, if the product quality decays faster (i.e., $k$ becomes larger), the likelihood that the retailer maximizes his freshness-keeping effort increases. On the contrary, if $c_{{\text{f}}} > kp$ or $T_{{\text{L}}} \ge A$, no freshness-keeping effort is advisable.

### The optimal $t_{{\text{d}}}^{*}$

The optimal rationing time $t_{{\text{d}}}^{*}$ for optimizing (15) cannot be analytically examined, due to the complexity in two aspects: the inventory and demand dynamics (as given in Table 2), and the conditions of the optimal freshness-keeping effort $u ^{*}$. Therefore, in this section, we develop the following algorithm to investigate the optimal decision $t_{d}^{*}$ for Q1 and Q2.

Step 1. Start with $$ i = 1$$, and initialize $$ T\left( i \right) = 1$$.

Step 2. For $0 < t_{{\text{d}}} \le T\left( i \right)$: search $t_{{{\text{d1}}}}^{*}$ for maximizing $\Pi^{{{\text{D}}\& {\text{N}}}}$ given in (13), and $t_{{{\text{d2}}}}^{*}$ for maximizing $\Pi^{{{\text{N}}\& {\text{D}}}}$ given in (14).

Step 3. $t_{{\text{d}}}^{*} = t_{{{\text{d1}}}}^{*}$ and the optimal strategy type is determined as “D&N” if $\Pi^{{{\text{D}}\& {\text{N}}}} \left( {t_{{{\text{d1}}}}^{*} } \right) > \Pi^{{{\text{N}}\& {\text{D}}}} \left( {t_{{{\text{d}}2}}^{*} } \right)$. Otherwise, $t_{{\text{d}}}^{*} = t_{{{\text{d2}}}}^{*}$ and the optimal strategy type is “N &D”.

Step 4. Set $i = i + 1$ and go back to Step 2 until $T\left( i \right) \ge T_{\max }$. The optimal $t_{{\text{d}}}^{*}$ for the disruption with length $T$ is determined.

Next, based on the proposed algorithm, we visually examine the optimal rationing time $t_{{\text{d}}}^{*}$ and the corresponding strategy type (“N&D” or “D&N”) for different disruptions. We also generate further insights into the roles of relative factors such as $c_{{\text{f}}}$*,* $T_{{\text{L}}}$*,* $r$*,* $\theta$*,* and $k$. To this end, we establish a basic setting as follows: $p = 100$, $d_{0} = 100$, $c_{{\text{h}}} = 1$,$c_{{\text{r}}} = 10$,$c_{{\text{f}}} = 20$, $T_{{\text{L}}} = 10$, $\theta = 0.1$, $r = 5$, $k = 0.5$, and $T_{{{\text{max}}}} = 30$. Then, we let the relevant parameters vary, and observe how the optimal delivery time $t_{{\text{d}}}^{*}$ will change. Note that, we focus on the variation trends of relative factors and have run abundant analysis on other basic values of these factors. Our main findings derived from the given setting would not change in the following.

**Fig. 3:**
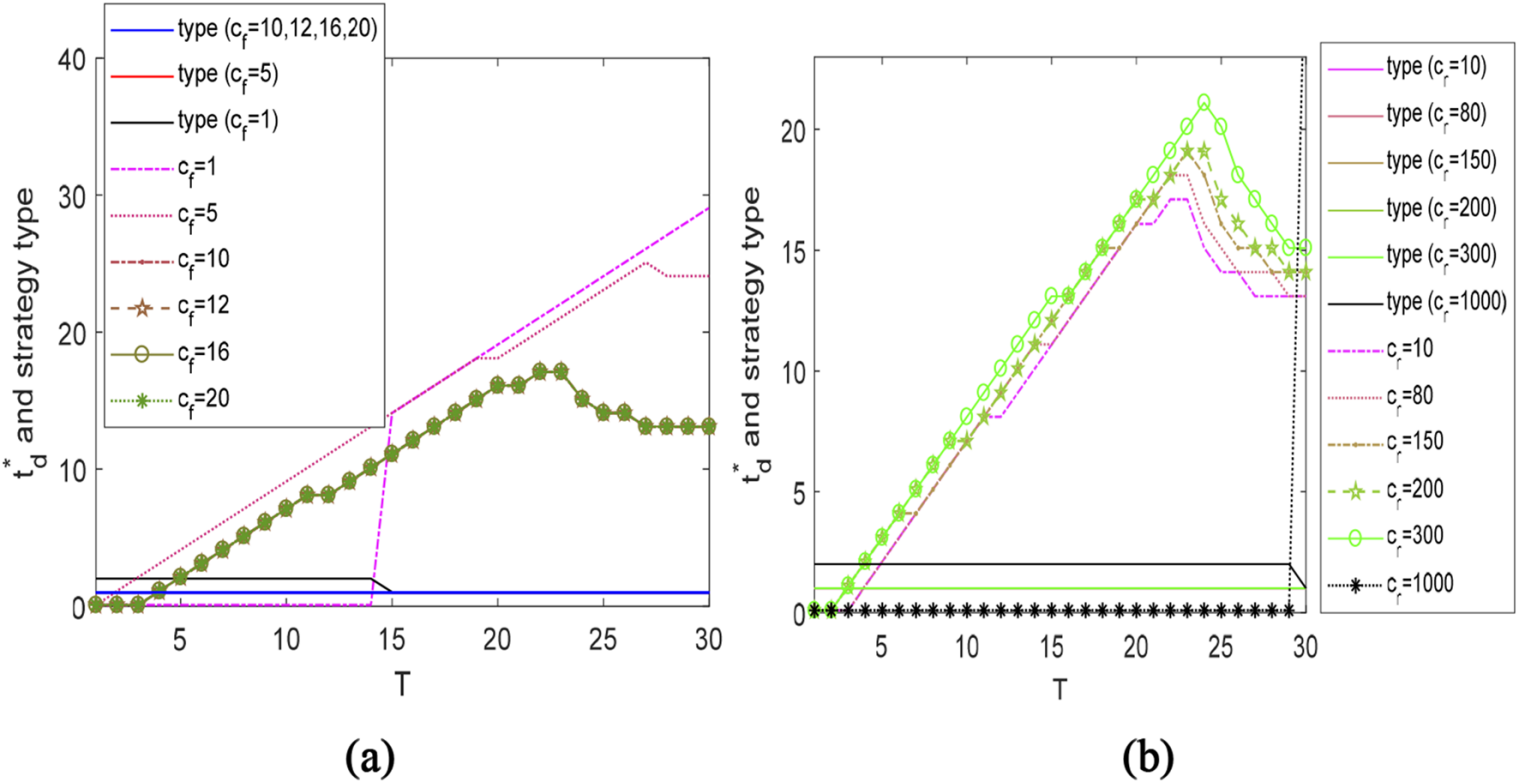
The optimal rationing time $t_{{\text{d}}}^{*}$ and strategy types under different $c_{{\text{f}}}$ and $c_{{\text{r}}}$

**Fig. 4:**
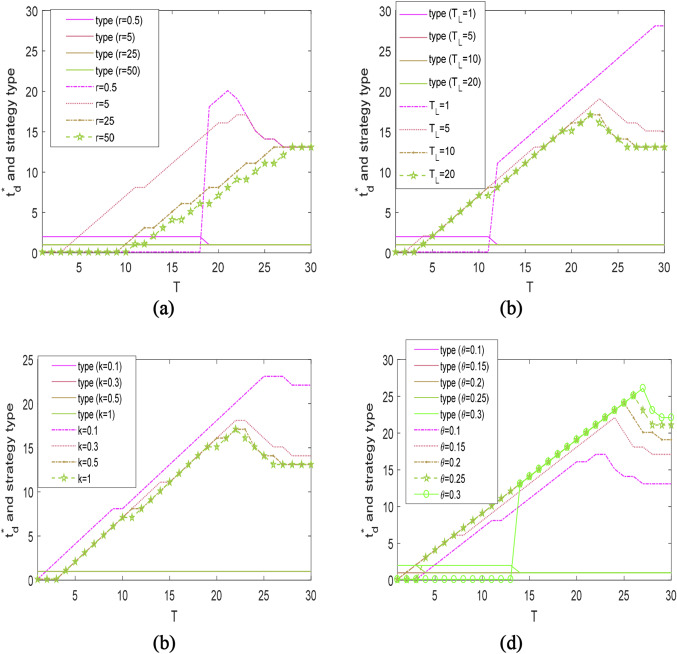
The optimal rationing time $t_{{\text{d}}}^{*}$ and strategy type under different $r$, $T_{L}$, $k$, and $\theta$, while $c_{{\text{f}}} = 20$

**Fig. 5:**
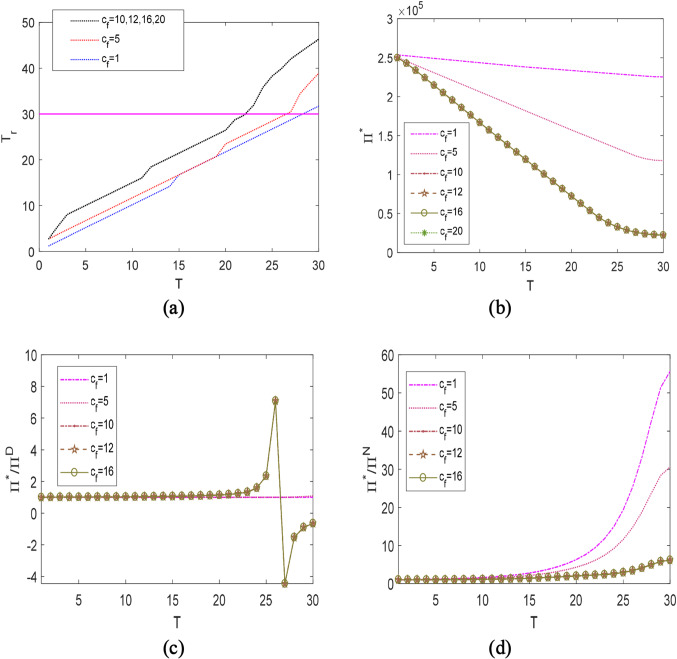
The $T_{{\text{r}}}$ (**a**) and the value of strategic rationing (**b**–**d**), under different $c_{{\text{f}}}$

Unless otherwise stated, in Figs. 3, 4, 5, the result of “${\text{type}} = 1$” refers to the strategy “D&N”, “${\text{type}} = 2$” stands for the strategy “N&D”. Furthermore, together with Table 3, the strategy “D” is represented by the result of “${\text{type}} = 1, t_{{\text{d}}}^{*} = T$” or “${\text{type}} = 2,\; t_{{\text{d}}}^{*} \approx 0$”, and the strategy “N” corresponds to “${\text{type}} = 1, \;t_{{\text{d}}}^{*} \approx 0$” or “${\text{type}} = 2,$ $t_{{\text{d}}}^{*} = T$”.

### The $t_{{\text{d}}}^{*}$ under different values of $c_{{\text{f}}}$ and $c_{{\text{r}}}$

Figure 3a shows the optimal rationing time $t_{{\text{d}}}^{*}$ and the strategy type (“D&N” or “N&D”) to cope with disruptions of different lengths, under different freshness-keeping prices $c_{{\text{f}}}$. The *x*-axis represents the disruption length. The results indicate that $t_{{\text{d}}}^{*}$ falls into two patterns based on the value of $c_{{\text{f}}}$: pattern 1 where $c_{{\text{f}}} < 10$ and pattern 2 where $c_{{\text{f}}} \ge 10$. The reason behind these two patterns is linked to the optimal freshness-keeping effort $u^{*}$. According to the results provided in Proposition 1, we find: (a) if $c_{{\text{f}}}$ exceeds $9.93$ under the given setting, freshness-keeping is inadvisable; (b) otherwise, the maximum freshness-keeping effort is suggested. In other words, the freshness-keeping efforts under these two patterns are determined as: $u^{*} = 1$ in Pattern 1; $u^{*} = 0$ in Pattern 2.

With $u^{*} = 1$ in Pattern 1, $t_{{\text{d}}}^{*}$ exhibits the following trends. If $c_{{\text{f}}}$ is small enough (e.g., $c_{{\text{f}}} = 1$), it is optimal to arrange deliveries to all customers during disruption, that is, adopting “D” strategy. If $c_{{\text{f}}}$ becomes relatively large (e.g., $c_{{\text{f}}} = 5$), the retailer should adjust the strategy type in accordance with the disruption length. The strategy “D” is still advantageous to hedge against short disruptions. However, for long disruptions, the strategy “D&N” is of superiority. To be specific, to avoid the negative impact, such as the devastating negative word-of-mouth facilitated by a large number of customers who have experienced long periods of out-of-stock, we suggest the following tactic for the retailer: deliver the products to the customers who are willing to purchase during the early period of the disruption; then, announce stock-outs to the second batch of customers arriving later.

With $u^{*} = 0$ in Pattern 2, no freshness-keeping effort is taken, thus $t_{{\text{d}}}^{*}$ barely changes with $c_{{\text{f}}}$. As a consequence of the absence of such preservation in product quality, the retailer significantly adjusts $t_{{\text{d}}}^{*}$ to manage different lengths of disruptions. It is preferable to implement the non-delivery strategy “N” for short disruptions, and the strategy “D&N” for medium and long disruptions. It is worth noting, long disruptions require a smaller $t_{{\text{d}}}^{*}$ than medium disruptions. The result reveals an interesting finding. Rather than arrange more deliveries, we suggest that the retailer creates unsatisfied demand earlier in mitigating longer disruptions. This non-intuitive result is reasonable. In view of the attractive recovery cost ($c_{{\text{r}}} = 10$) and the fact that the demand rate will not drop dramatically during the information propagation of stock-outs ($\theta = 0.1$), the decision that fulfills demands and pays a huge penalty for quality loss is inferior to the following countermeasure: create unsatisfied demand during the disruption, then gradually recover the market potential by reducing the customers’ dissatisfaction through incentive policies after the disruption ends. The critical time $T_{{\text{r}}}$ when the market can be fully recovered to the pre-disruption level is shown in Fig. 5a. Note, the above observations might not hold under different values of $c_{r}$ and $\theta$. Thus, further clarifications are presented in Figs. 3b and 4b.

Figure 3b depicts the variation trend of $t_{{\text{d}}}^{*}$ with the demand recovery cost $c_{{\text{r}}}$. A larger $c_{{\text{r}}}$ leads to a larger $t_{{\text{d}}}^{*}$ in hedging against long disruptions. In other words, if it requires a large cost to recover the lost market, the retailer should directly reduce the creation of unsatisfied demand during the disruption, by lengthening the time interval of adopting “D” strategy. Clearly, with this trend, the strategy “D” should be implemented during the entire disrupted period when $c_{{\text{r}}}$ exceeds a threshold (e.g., $c_{{\text{r}}} \ge 1000$ in Fig. 3b).

### The $t_{{\text{d}}}^{*}$ under different values of $r$, $T_{{\text{L}}}$, $k$, and $\theta$

Figure 4 shows how $t_{{\text{d}}}^{*}$ changes with respect to the demand recovery time $r$, the delivery lead time $T_{{\text{L}}}$, the quality decay rate $k$, and the customers’ learning intensity $\theta$.

As indicated in Fig. 4a, in hedging against short disruptions, it is optimal to employ the strategy “D” when $r$ is extremely small, and the strategy “N” otherwise. In other words, if it takes an essentially long time for the damaged market to be fully recovered, the retailer should fulfill all demands during the disruption. Conversely, it is preferable to create unsatisfied demand during the entire transportation disruption. For medium and long disruptions, it is optimal to implement “D&N”. The difference is that when mitigating medium disruptions, $t_{{\text{d}}}^{*}$ becomes smaller as the recovery speed becomes larger. However, if the disruption lasts for a long time, $t_{{\text{d}}}^{*}$ will not change with $r$.

We also observe from Fig. 4b–d that $t_{{\text{d}}}^{*}$ under the strategy “D&N” becomes smaller, if one of the following situations happens: the delivery lead time becomes longer, the quality decays quicker, or the customers’ learning intensity is smaller. In particular, $t_{{\text{d}}}^{*}$ will not change if $k$ or $T_{{\text{L}}}$ exceeds a critical value. The reason is intuitive considering that the product quality will approach zero for a relatively large $k$ or $T_{{\text{L}}}$. It is also worth noting that if the customers’ learning intensity becomes large enough, rationing is disadvantageous to hedge against any long disruptions, that is, the optimal strategy switches from “D&N” to “D”.

All in all, we also find that the “N&D” strategy is never profitable. The reason is intuitive. There are several kinds of losses (costs) incurred during the creation of rationing, including the future demand loss driven by negative word-of-mouth, the inventory holding cost, and the lost sales. Thereby, if it is necessary to create a fraction of unsatisfied demands during disruption, it is better to create them in the late period.

### The value of strategic rationing

To present the value of strategic rationing, we address a comparison analysis between our proposed strategies incorporating rationing and other pure strategies without rationing. To this end, Fig. 5b–d depict the ratios of ${\Pi }^{*}$ to ${\Pi }^{D}$ and ${\Pi }^{*}$ to ${\Pi }^{N}$. Where, ${\Pi }^{D}$ and ${\Pi }^{N}$ give the profits of the two commonly utilized strategies without strategic rationing, i.e., the full-delivery “D” and the non-delivery “N”. ${\Pi }^{*}$ represents the profit of our proposed selling strategy (i.e., strategic rationing).

Two findings are observed. First, by strategic rationing, the retailer can raise its profit by up to 55 times (see Fig. 5d). Second, the profit gaining through the optimal rationing increases with respect to the disruption length. Without rationing, the retailer might achieve a negative profit, if he insists on delivering products to all customers during long disruptions (see Fig. 5b, c). The observations further confirm the importance of strategic rationing, when hedging against long disruptions.

## Conclusions

In this paper, we consider a retailer selling perishable fresh products through an online channel, where customers could directly learn the purchase information of the preceding periods. An unexpected event occurs leading to a transportation interruption, during which the delivery lead time extends. The optimal selling strategies that determine rationing time and freshness-keeping effort are explored for the retailer to mitigate disruption impacts.

The disruption impact is evaluated from three periods: disruption duration, delivery lead time, and disruption recovery. During the transportation disruption, the cost of creating rationing (i.e., creating unsatisfied demand on purpose) not only considers lost sales, but also takes into account future demand loss facilitated from negative word-of-mouth (i.e., demand learning). In the absence of rationing, freshness-keeping cost and quality-dependent compensation are induced due to the long delivery lead time. After the transportation disruption ends, the lost demand could be fully or partially recovered by employing incentives at a certain financial cost and time.

Based on these considerations, we analytically present the post-disruption dynamics of demand and inventory. Afterward, a model is formulated to identify the joint decision of freshness-keeping effort and rationing time, with the objective of maximizing the post-disruption profit. The optimal freshness-keeping effort is presented in closed form. The results suggest that the retailer inputs the greatest freshness-keeping efforts to preserve the quality of fresh products, under one of the following circumstances: the selling price of the fresh products is high, or the freshness-keeping price is low while the delivery lead time is short.

Lastly, according to the critical point in time of creating rationing, we propose three optimal selling strategies by a numerical analysis: a full-delivery strategy “D”, a non-delivery strategy “N”, and a mixed strategy “D&N” that only arranges deliveries at the first phase. We also provide some key managerial insights for practitioners to hedge against different lengths of disruptions under different circumstances. In general, the non-delivery strategy “N” only suffices to alleviate short disruptions in the following situations: the freshness-keeping price or the demand recovery speed is large, and the succeeding customers’ purchase willingness will not be dramatically affected by the information generated from the formers. It is advisable to utilize the full-delivery strategy “D” if one of the occasions occurs: the lost demand is difficult to be recovered, the disruption lasts short while the freshness-keeping price is attractive, or the customers’ learning tendency towards word-of-mouth (information) is large. Except for the above special circumstances, the “D&N” strategy is superior. Further suggestions are also presented to indicate how the relative factors play different roles in changing the rationing period of “D&N”. For example, when employing “D&N” to cope with the disruption, rationing should be created earlier under one of the following events: a smaller recovery cost or customers’ learning intensity towards information; a larger delivery lead time or quality decay rate.

This study raises several directions for future work. For example, we focus on a single deterministic disruption during a single selling period, in a single-tier supply chain composed of a single retailer and multiple customers. The model established on a basic setting could be a baseline. Therefore, a direct direction worthy of exploration is to extend the question regarding multiple echelons, random disruptions, and multiple selling periods. On the other hand, in the present paper, we assume that retailers are committed to restoring the lost market after the outage ends. Another idea is to incorporate recovery decisions during the disruption.

## Figures and Tables

**Fig. 6:**
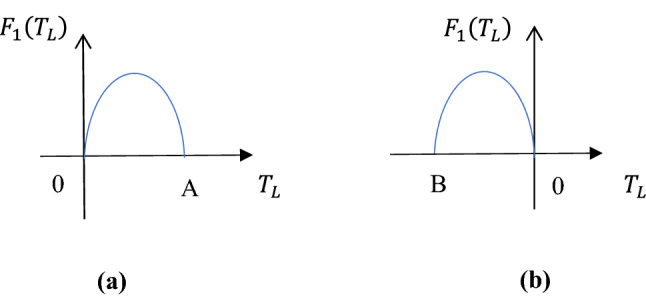
The function $\left( {1 - {\text{e}}^{{ - kT_{{\text{L}}} }} } \right)p - c_{{\text{f}}} T_{{\text{L}}}$ with respect to $T_{{\text{L}}}$

## Appendix

### Proof of Proposition 1

(i) Under the “D&N” strategy: According to $\Pi^{{{\text{D}}\& {\text{N}}}}$ given in (13), the first-order and the second-order derivatives of $\Pi^{{{\text{D}}\& {\text{N}}}}$ with respect to the decision variable $u$ is given as
  17 $$ \begin{gathered} \frac{{\partial \Pi^{{{\text{D}}\& {\text{N}}}} { }}}{\partial u} = - \frac{{\partial \left[ {c_{{\text{f}}} uT_{{\text{L}}} + \left( {1 - q\left( u \right)} \right)p} \right]{ }}}{\partial u}\mathop \int \limits_{0}^{{t_{{\text{d}}} }} d_{0} {\text{d}}t \hfill \\ = - \frac{{\partial \left[ {c_{{\text{f}}} uT_{{\text{L}}} + \left( {1 - {\text{e}}^{{ - k\left( {1 - u} \right)T_{{\text{L}}} }} } \right)p} \right]{ }}}{\partial u}\mathop \int \limits_{0}^{{t_{d} }} d_{0} {\text{d}}t \hfill \\ = - \left[ {c_{{\text{f}}} - kp{\text{e}}^{{ - k\left( {1 - u} \right)T_{{\text{L}}} }} } \right]T_{{\text{L}}} \mathop \int \limits_{0}^{{t_{{\text{d}}} }} d_{0} {\text{d}}t, \hfill \\ \end{gathered} $$
  18 $$ \frac{{\partial^{2} \Pi^{{{\text{D}}\& {\text{N}}}} { }}}{{\partial u^{2} }} = k^{2} pT_{{\text{L}}}^{2} {\text{e}}^{{ - k\left( {1 - u} \right)T_{{\text{L}}} }} \mathop \int \limits_{0}^{{t_{{\text{d}}} }} d_{0} {\text{d}}t > 0. $$

Note that, $$t_{{\text{d}}} > 0$$. As indicated from (17) and (18), $$\Pi^{{{\text{D}}\& {\text{N}}}}$$ is concave with $$u$$. Thus, the optimal freshness-keeping effort $$u^{*}$$ to maximize the retailer’s profit $$\Pi^{{{\text{D}}\& {\text{N}}}}$$ is achieved at $$u^{*} = 1 $$ or $$u^{*} = 0$$.

Next, by comparing $\left. {\Pi^{{{\text{D}}\& {\text{N}}}} } \right|_{{u^{*} = 1}}$ with $\left. {\Pi^{{{\text{D}}\& {\text{N}}}} } \right|_{{u^{*} = 0}}$, we see that

19 $$ \left. {\Pi^{{{\text{D}}\& {\text{N}}}} } \right|_{{u^{*} = 1}} - \left. {\Pi^{{{\text{D}}\& {\text{N}}}} } \right|_{{u^{*} = 0}} = \left[ { - c_{{\text{f}}} T_{{\text{L}}} + \left( {1 - {\text{e}}^{{ - kT_{{\text{L}}} }} } \right)p} \right]\mathop \int \limits_{0}^{{t_{{\text{d}}} }} d_{0} {\text{d}}t. $$

Equation (19) indicates that the optimal decision is $u^{*} = 1$ if $\left( {1 - {\text{e}}^{{ - kT_{{\text{L}}} }} } \right)p - c_{{\text{f}}} T_{{\text{L}}} > 0$ and $u^{*} = 0$ otherwise.

(ii) Under the “N&D” strategy: According to $\Pi^{N\& D}$, we have
  20 $$ \frac{{\partial \Pi^{{{\text{N}}\& {\text{D}}}} { }}}{\partial u} = - \left[ {c_{{\text{f}}} - kp{\text{e}}^{{k\left( {1 - u} \right)T_{{\text{L}}} }} } \right]T_{{\text{L}}} \mathop \int \limits_{{t_{{\text{d}}} }}^{T} d_{1}^{{{\text{N}}\& {\text{D}}}} \left( {t_{{\text{d}}} } \right){\text{d}}t. $$

Note that, no shipment is realized during the disruption if $t_{{\text{d}}} = T$. In other words, there is no need to input any freshness-keeping effort. Thus, we focus on discussing $u^{*}$ under $0 < t_{{\text{d}}} < T$. Repeating the analysis of (17)–(19), we can directly confirm that the result of $u^{*}$ also hold for this case.

Summing up, Proposition 1 is achieved.

### Proof of Corollary 1

For the convenience of the statement, we denote the left-hand side of (16) as a function with respect to $T_{L}$.

21 $$ F_{1} \left( {T_{{\text{L}}} } \right) = \left( {1 - {\text{e}}^{{ - kT_{{\text{L}}} }} } \right)p - c_{{\text{f}}} T_{{\text{L}}} $$

Give (21), we see that

22 $$ \begin{gathered} \frac{{d\left[ {F_{1} \left( {T_{{\text{L}}} } \right)} \right]}}{{dT_{{\text{L}}} }} = kp{\text{e}}^{{ - kT_{{\text{L}}} }} - c_{{\text{f}}} \;{\text{and}} \hfill \\ \frac{{d^{2} \left[ {F_{1} \left( {T_{{\text{L}}} } \right)} \right]}}{{dT_{{\text{L}}}^{2} }} = - k^{2} p{\text{e}}^{{ - kT_{{\text{L}}} }} < 0. \hfill \\ \end{gathered} $$

Equation (22) indicates that $F_{1} \left( {T_{{\text{L}}} } \right)$ is convex with $T_{{\text{L}}}$. On the other hand, according to (21), we see that $F_{1} \left( {T_{{\text{L}}} } \right) = 0$ when $T_{{\text{L}}} = 0$. Therefore, we can conclude that there is another critical value of $T_{{\text{L}}}$ such that $F_{1} \left( {T_{{\text{L}}} } \right) = 0$, as shown in Fig. 6.

(i) If $\frac{{d\left[ {F_{1} \left( {T_{{\text{L}}} } \right)} \right]}}{{dT_{{\text{L}}} }}\left. \right|_{{T_{{\text{L}}} = 0}} > 0$, that is, if $kp - c_{{\text{f}}} > 0$: The curve of $F_{1} \left( {T_{{\text{L}}} } \right)$ is depicted in Fig. 6a. Under this situation, we find that $F_{1} \left( {T_{L} } \right) > 0$ holds if and only if $0 < T_{{\text{L}}} < A$.
(ii) Otherwise, if $kp - c_{{\text{f}}} < 0$: The curve of $F_{1} \left( {T_{{\text{L}}} } \right)$ is depicted in Fig. 6b. The conclusion can be drawn that $F_{1} \left( {T_{L} } \right) \le 0$ holds for all $T_{{\text{L}}} > 0$.

Corollary 1 is achieved.
